# Acute effects of single and repeated mild traumatic brain injury on levels of neurometabolites, lipids, and mitochondrial function in male rats

**DOI:** 10.3389/fnmol.2023.1208697

**Published:** 2023-06-29

**Authors:** Josh Allen, Louise Pham, Simon T. Bond, William T. O’Brien, Gershon Spitz, Sandy R. Shultz, Brian G. Drew, David K. Wright, Stuart J. McDonald

**Affiliations:** ^1^Department of Neuroscience, Central Clinical School, Monash University, Melbourne, VIC, Australia; ^2^Department of Diabetes, Central Clinical School, Monash University, Melbourne, VIC, Australia; ^3^Baker Heart & Diabetes Institute, Melbourne, VIC, Australia; ^4^Baker Department of Cardiometabolic Health, University of Melbourne, Melbourne, VIC, Australia; ^5^Monash-Epworth Rehabilitation Research Centre, Turner Institute for Brain and Mental Health, School of Psychological Sciences, Monash University, Melbourne, VIC, Australia; ^6^Health Sciences, Vancouver Island University, Nanaimo, BC, Canada; ^7^Department of Medicine, University of Melbourne, Parkville, VIC, Australia

**Keywords:** concussion, proton magnetic resonance spectroscopy, MRI, lipids, mitochondria, biomarker, behavior

## Abstract

**Introduction:**

Mild traumatic brain injuries (mTBIs) are the most common form of acquired brain injury. Symptoms of mTBI are thought to be associated with a neuropathological cascade, potentially involving the dysregulation of neurometabolites, lipids, and mitochondrial bioenergetics. Such alterations may play a role in the period of enhanced vulnerability that occurs after mTBI, such that a second mTBI will exacerbate neuropathology. However, it is unclear whether mTBI-induced alterations in neurometabolites and lipids that are involved in energy metabolism and other important cellular functions are exacerbated by repeat mTBI, and if such alterations are associated with mitochondrial dysfunction.

**Methods:**

In this experiment, using a well-established awake-closed head injury (ACHI) paradigm to model mTBI, male rats were subjected to a single injury, or five injuries delivered 1 day apart, and injuries were confirmed with a beam-walk task and a video observation protocol. Abundance of several neurometabolites was evaluated 24 h post-final injury in the ipsilateral and contralateral hippocampus using *in vivo* proton magnetic resonance spectroscopy (1H-MRS), and mitochondrial bioenergetics were evaluated 30 h post-final injury, or at 24 h in place of 1H-MRS, in the rostral half of the ipsilateral hippocampus. Lipidomic evaluations were conducted in the ipsilateral hippocampus and cortex.

**Results:**

We found that behavioral deficits in the beam task persisted 1- and 4 h after the final injury in rats that received repetitive mTBIs, and this was paralleled by an increase and decrease in hippocampal glutamine and glucose, respectively, whereas a single mTBI had no effect on sensorimotor and metabolic measurements. No group differences were observed in lipid levels and mitochondrial bioenergetics in the hippocampus, although some lipids were altered in the cortex after repeated mTBI.

**Discussion:**

The decrease in performance in sensorimotor tests and the presence of more neurometabolic and lipidomic abnormalities, after repeated but not singular mTBI, indicates that multiple concussions in short succession can have cumulative effects. Further preclinical research efforts are required to understand the underlying mechanisms that drive these alterations to establish biomarkers and inform treatment strategies to improve patient outcomes.

## Introduction

Mild traumatic brain injury (mTBI), often referred to as concussion, is a serious medical issue with a 10-fold higher burden on the healthcare system than both moderate and severe traumatic brain injuries (TBI) (Corwin et al., 2017; Dewan et al., 2019). mTBIs are heterogeneous in nature and can result in a variety of neurobehavioral sequalae. Acute symptoms of mTBI include but are not limited to disorientation, dizziness, headaches, and emotional, sensorimotor, and cognitive deficits (Schimmel et al., 2017). These symptoms are thought to be caused by an acute and typically transient neuropathophysiological cascade (Pavlova et al., 2018). Evidence suggests that mTBI induces glutamatergic-driven excitotoxicity, neurometabolic and lipid imbalances, and a mitochondrial energy crisis (Ng and Lee, 2019; Romeu-Mejia et al., 2019; Khatri et al., 2021). Furthermore, pharmacological modulators of metabolic derangement and mitochondrial dysfunction, such as excitotoxicity-dampening compounds or antioxidants, are protective against brain trauma (Hwabejire et al., 2013; Ismail et al., 2020). Repeated mTBIs that occur in short succession exacerbate neuropathology and symptomology (Miller et al., 2013; Dretsch et al., 2015), and there is growing evidence that repeated mTBIs increase the risk of developing progressive neurodegenerative and emotional disorders, including Alzheimer’s disease, amyotrophic lateral sclerosis, chronic traumatic encephalopathy, depression, anxiety, and epilepsy (Abel, 2007; Graham and Sharp, 2019; Asken and Rabinovici, 2021). However, despite increased research efforts on the effects of mTBI, there are still important knowledge gaps surrounding the cellular consequences of singular and repeated mTBIs. Uncovering specific cellular alterations after mTBI (i.e., biomarkers) could inform personalized treatment approaches.

One recognised and potentially prominent neuropathological aspect of the mTBI cascade is neurometabolic dysfunction (Kim et al., 2017; Veeramuthu et al., 2018), which can be measured in brain tissue via ^1^H-MRS. Several neurometabolites, such as N-acetylasparate (NAA), Creatine (Cr), phosphocreatine (PCr), glucose (Glc), glutamate (Glu), and glutamine (Gln), have been identified to have putative utility in assisting mTBI diagnosis and prognosis (Eisele et al., 2020). Cellular damage induced by mTBI can lead to the excessive release of Glu and thereafter neuronal excitotoxicity and potentially cell death (Baracaldo-Santamaría et al., 2022). Alterations in the concentrations of Glu and Gln, the storage form of Glu (Dorsett et al., 2017; Baracaldo-Santamaría et al., 2022), are indicative of altered brain metabolism, but changes in their abundance have previously been reported as a sum (Ashwal et al., 2004; Shutter et al., 2004; Yeo et al., 2011). Using advanced ^1^H-MRS, one can gather more accurate measurements by evaluating Glu and Gln levels separately (Ramadan et al., 2013). Alterations in the levels of Glc, which could lead to hyper-glycolysis (Vagnozzi et al., 2008), has been postulated to be involved in TBI (Yuan et al., 2022), but investigations to answer this question with the use of ^1^H-MRS are also lacking. NAA has been flagged as a potential biomarker of neuronal integrity and mitochondrial function, and alterations in NAA/Cr may represent a diagnostic biomarker for mTBI (Vagnozzi et al., 2005). A clinical study found that NAA/Cr alterations and recovery post-mTBI worsened when a second injury had occurred shortly after the initial injury; notably, metabolic alterations persisted beyond symptom resolution (Vagnozzi et al., 2008). In addition, Cr and PCr are thought to be an indicator of cellular energetics (Wallimann et al., 2011) and are found abundantly in the brain (Stovell et al., 2017), where they are significantly altered in response to TBI (Gasparovic et al., 2009; Signoretti et al., 2010; Yeo et al., 2011). Another metabolite, choline, is a major component of phospholipid membranes, and the elevation of glycerolphosphocholine and phosphocholine (GPC and PCh) after TBI are thought to indicate cell membrane breakdown (Shutter et al., 2004). Altogether, it has been speculated that mTBI-induced metabolic changes are, at least partially, responsible for a transient epoch of enhanced vulnerability to cerebral insults – with shorter inter-injury intervals leading to increased neurometabolic deficits (Vagnozzi et al., 2007, 2008).

Mitochondria are vital for the regulation of cellular metabolism due to their roles in ATP production and intracellular calcium buffering (Severo et al., 2020). mTBI can lead to the excessive influx of calcium into cells, which is sequestered into mitochondria and instigates mitochondrial allostatic overload and consequently, ionic imbalances, an accumulation of harmful reactive oxygen species, loss of membrane potential, and deficient ATP production (Hubbard et al., 2018). Furthermore, mitochondrial dysfunction is observed in patients with disorders that are co-morbid with mTBI, like depression, anxiety, cognitive deficits, and epilepsy, suggesting that mitochondrial impairments represent a vulnerability factor for subsequent pathological disturbances (Singh et al., 2021). Nevertheless, it is unclear how mitochondrial deficits after mTBIs are linked to ongoing or progressive neurometabolic and lipidomic alterations.

Another potentially prominent neuropathological characteristic of mTBI is the dysregulation of lipids, which are crucial to the structural and functional integrity of neuronal and non-neuronal cells, as well as cellular organelles, such as mitochondria (Ojo et al., 2019). Examples of lipids that may be implicated in mTBI include phosphatidylethanolamine (PE), Bis(monoacylglycero)phosphate (BMP), acylcarnitine (AC & AC-OH), cardiolipin (CL), lysophosphatidylcholines (LPC), lysophosphatidylethanolamine (LPE), and lysophosphatidylinositol (LPI). PE is a major component of cell membranes that is implicated in membrane stability, trafficking, permeability, and fluidity (Yoon et al., 2022), and it has also been linked to neurodegenerative disorders (Anyaegbu et al., 2022). BMP is enriched in the lysosomal network and is suspected to represent a biomarker of phagocytizing macrophages/microglia in brain tissue and is implicated in various physiological cellular functions, such as cell signaling and the regulation of neuronal growth (Nielsen et al., 2016). Acylcarnitines are generated from the conjugation of acyl-coenzyme A (CoA) with carnitine, to facilitate the transport of fatty acids across the mitochondrial membrane from the cytosol into the mitochondrial matrix, for β oxidation and cellular energy production, and are available as supplements with putative neuroprotective effects (Allen et al., 2021). CL makes up 20% of the inner mitochondrial membrane and are essential for the optimization of enzymes involved in mitochondrial bioenergetics, as well as fusion and fission processes, and apoptosis, with CL oxidization being observed before the appearance of apoptotic biomarkers after TBI in rats (Bayir et al., 2007; Paradies et al., 2014). LPCs are molecules derived from the degradation of phospholipids in the cell membrane, and, once released by apoptotic cells, are thought to act as a “find-me” signal for phagocytes (Park and Kim, 2017) and may be an indicator of injury severity (Pasvogel et al., 2008, 2010). Another minor component of the cell membrane, LPE, is involved in the activation of enzymes and the mediation of cell signaling, and alterations in its expression in a mouse model of mTBI paralleled those detected in soldiers that had endured chronic mTBI (Emmerich et al., 2017), but alterations may be region-specific and time-sensitive (Ojo et al., 2018). LPI is a bioactive lipid that targets the GRP55 endocannabinoid receptor and influences cell growth, differentiation, and motility in various cell types (Calvillo-Robledo et al., 2022), and it was shown to be protective against glutamate-induced excitotoxicity (Blondeau et al., 2002). However, few research endeavours have evaluated the trajectory of lipid alterations after single and repeated mTBIs, and the relationship between lipid abnormalities and other pathological characteristics of mTBI, such as neurometabolic and mitochondrial dysfunction.

Therefore, the present study investigated the metabolic and lipidomic changes after single and repeated mTBI, and whether mitochondrial impairments are associated with mTBI-induced metabolic and lipidomic alterations.

## Methods

### Animal husbandry

Male Sprague Dawley rats (*n* = 48) were obtained from Monash Animal Research Platform (Clayton, Victoria, Australia) at postnatal day (PND) 25–27. Three rats were housed per cage with a 12 h:12 h light/dark cycle and food and water available *ad libitum*. All procedures were approved by the Alfred Medical Research and Educational Precinct Animal Ethics Committee (E/1832/2018/M) and were within the ARRIVE guidelines and the Australian code of practice for the use and care of animals for scientific purposes by the Australian National Health and Medical Research Council. The rats were given 2 days to habituate to their enclosures upon arrival to the facility. Thereafter, they were handled 4 days per week until the first sham or mTBI procedure on PND 38–41.

### Experimental procedures

**Figure 1:**
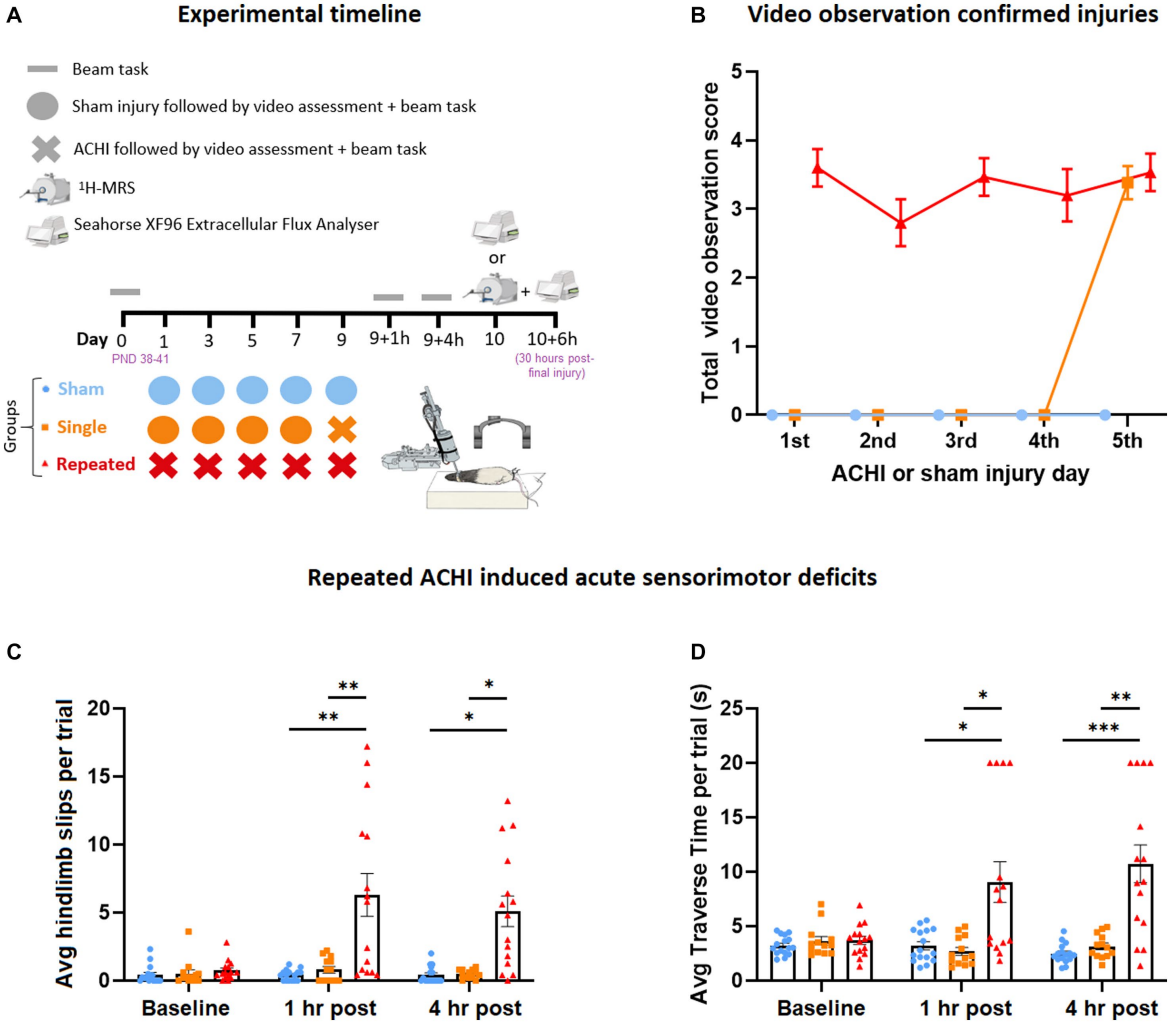
Effect of singular and repeated ACHIs at baseline, 1 h, and 4 h post-final injury. **(A)** Experimental timeline. **(B)** Video observation confirmed injuries. Repeated ACHIs increased the amount of beam hindlimb slips **(C)** and beam traverse time **(D)** compared to sham and singular ACHI. ^*^p < 0.05/^**^p < 0.01/^***^p < 0.001 sham or singular ACHI vs. repeated injury. Data are presented as mean ± SEM.

Rats (*n* = 16/group) were randomly assigned to receive either five sham mTBIs, four sham mTBIs followed by a single mTBI, or five mTBIs, with each procedure occurring 48 h apart (see experimental design in Figure 1A). mTBIs were modelled with an awake closed-head injury (ACHI) paradigm, which removes the confounding effects of surgery or anesthesia (Pham et al., 2019) – important because anesthetic agents can provide neuroprotection against brain injury (Burchell et al., 2013; Zhu et al., 2021) – and also allowed us to utilise a less confounded assessment of neurological signs of mTBI to confirm whether concussion-like symptoms were present immediately after the impact. Sham and ACHI procedures were completed similar to those described previously (Pham et al., 2019, 2021). Briefly, ACHI involved securing a steel helmet on the head of a restrained rat, with an impact site centered over the left parietal bone. The rat was then placed on a foam bed with the impact site positioned under a 5 mm tip that is attached to a controlled cortical impactor (Leica Biosystems, IL, United States). The helmets were struck by the impactor at a velocity of 6.5 m/s, at a depth of 4 mm, and with a dwell time of 100 ms, which forces the rat’s unrestrained head downward into the foam platform. Three rats were excluded from the study due to machine error during the procedure. The total duration of the procedure was less than 60 s. Sham injuries were performed identically to ACHI but the impact was triggered adjacent to the animal’s head. No skull fractures were observed during post-mortem analyses.

### Neurobehavioral testing to confirm mTBI

The absence of anaesthesia during the ACHI allowed for inspection of visual signs of TBI. We incorporated a scoring method for video signs of mTBI based on the recent injury definitions provided by the international consensus of video signs of concussion in professional sports (Davis et al., 2019). Researchers that were blinded to experimental groups gave rats a point if they showed video signs of mTBI, including lying motionless >2 s, visible signs of seizure or tonic posturing, impaired limb extension reflexes, body sprawling, and motor incoordination on the beam, as a clinically translatable confirmation of injury, with a maximum score of 5.

Once rats had self-righted and were mobile (typically within 60 s of injury), they were immediately exposed to an abbreviated beam task as another confirmation of injury. A full beam task was used to assess sensorimotor function at baseline (1 day prior to the first injury) and 1- and 4 h post-final injury. The rats were trained to traverse the beam 3 days prior to the first sham/ACHI; they were required to cross a 4 cm-wide beam for two consecutive trials at a distance of 10 cm, 25 cm, and 50 cm. This training process was repeated on a 2 cm-wide beam. During the beam test, rats crossed 50 cm of a wooden beam that was 2 cm-wide, and elevated 75 cm above the ground, with a protective mattress placed below to soften any falls. At the start-end of the beam there was a bright light (i.e., an aversive stimulus), and at the finish-end there was a darkened bedded “home cage” to encourage task completion. In a small number of cases, trials were re-performed if rats were immobile for longer than 5 seconds. Rats were given a maximal score of 20 s if they had not crossed the beam in this time, or if they fell off the beam, or exhibited two hindlimb slips. Beam slip data from a subset of these rats has been presented previously (O’Brien et al., 2021).

### Proton magnetic resonance spectroscopy

Twenty-four hours after the final injury, *in vivo* ^1^H-MRS was performed using a 9.4 T Bruker MRI (Bruker^™^ BioSpin^®^, United States). Rats were anaesthetised with 5% isoflurane inhalation and anesthesia was maintained throughout the 50–60 min procedure with 2% isoflurane inhalation using a nose cone. Body temperature was maintained with a heating pad connected to a water heating circulation system built into the rat cradle. Point Resolved Spectroscopy was acquired in the ipsilateral and contralateral hippocampus with VAPOR water suppression and outer volume saturation. An actively-decoupled, cryogenically-cooled, surface coil was used together with a volume resonator for excitation. Acquisition parameters were: repetition time = 2,500 ms; echo time = 16.5 ms; number of excitations = 256; voxel size = 2.5 × 1.5 × 2.4 mm^3^; and 4,096 points over 11 ppm. A non-water suppressed spectra was also acquired for absolute quantification of metabolite concentrations relative to water. LCModel was used to process ^1^H-MRS data with a metabolite basis set matched to field strength and echo time. The current study used absolute concentrations to quantify neurometabolites Glu, Gln, Glc, NAA, Cr + PCr, and GPC + PCh. Only spectra with a %-standard deviation of less than 20 were included in the analyses.

### Mitochondrial isolation

To isolate mitochondria, rats were anesthetized via isoflurane inhalation and injected intraperitoneally with Lethobarb (80 mg/kg) and decapitated, either 24- (no ^1^H-MRS; *n* = 4/group) or 30 h (after ^1^H-MRS; *n* = 4–6/group) post-final injury. Thereafter, the rostral half of the ipsilateral hippocampus was dissected and immediately placed in ice-cold buffer A (220 mM Mannitol, 70 mM Sucrose, 20 mM HEPES, 2 mM Tris–HCl, 1 mM EDTA/EGTA, pH 7.2) + 0.4% (w/v) fatty acid free BSA and then homogenized with a glass Dounce homogenizer. The homogenate was centrifuged at 650 g for 5 min at 4°C and the pellet was discarded, and the supernatant transferred to a fresh tube. The supernatant was repeatedly centrifuged at 650 g for 5 min until very little material was pelleted. The supernatant was then transferred to a high-speed centrifuge tube and centrifuged at 10,000 g for 5 min and the supernatant was discarded, and the crude mitochondrial pellet resuspended in isolation buffer A. Mitochondria were re-pelleted by centrifuging at 10,000 g for 5 min, and the supernatant was discarded, and the pellet resuspended in 1 mL of isolation buffer B (220 mM Mannitol, 70 mM Sucrose, 10 mM Tris-HCl, 1 mM EDTA, pH 7.2). The final pellet was collected by centrifuging at 10,000 g for 5 min, discarding the supernatant and resuspending the pellet in isolation buffer B at a ratio of 1.5 mL per 1 g of original weight.

### Isolated mitochondrial respiration

To examine mitochondrial function and respiration, oxygen consumption in isolated mitochondrial preps was measured using an XFe-96 Seahorse Bioanalyzer (Agilent, United States) as previously described (Watt et al., 2021). Protein was quantified in fresh isolated mitochondrial preps from the hippocampus using BCA (Pierce Reagent kit). A total of 3 ug of isolated mitochondria in 180 μL MAS buffer (Sucrose 70 mM, Mannitol 220 mM, KH2PO4 5 mM, MgCl2 5 mM, HEPES 2 mM, EGTA 1 mM, BSA fatty acid free 0.2%, pH 7.4 adjusted with KOH 1 mol/L) was loaded into a 96 well seahorse cell culture plate (Seahorse XFe96 FluxPak) and centrifuged at 2000 g for 15 min to adhere the mitochondria to the bottom of the plate. To examine the degree of coupling between the electron transport chain (ETC) and oxidative phosphorylation, a coupling assay was performed. The coupling assay was used to determine basal, state 3 and state 4 respiration with sequential injections of ADP 3 mM, Oligomycin 1 μM, Carbonyl Cyanide-P-Trifluoromethoxyphenylhydrazone (FCCP) 1 μM, and Antimycin-A 4 μM. For the coupling assay, isolated mitochondria were adhered to 96 well plate in MAS media supplemented with Sodium Pyruvate 10 mM and Malate 10 mM.

To examine sequential electron flow through different complexes of the electron transport chain, an electron flow assay was performed. The electron flow assay was used to determine Complex I, Complex II, and Complex IV mediated respiration with sequential injections of Rotenone 2 μM, Succinate 10 mM, Antimycin-A 4 μM, and L-ascorbate 10 mM + N,N,N′,N′-Tetramethyl-P-Phenylenediamine (TMPD) 100 μM. For the electron flow assay, isolated mitochondria were adhered to 96 well plate in MAS media supplemented with Sodium Pyruvate 10 mM, Malate 10 mM, and FCCP 4 μM.

### Lipidomics

Lipidomics was analyzed in cortex and remaining half of hippocampus tissues from rats culled as described above in mitochondrial isolation method (*n* = 9–12/group). For Lipidomics analysis, tissues were homogenized in PBS and lipids were extracted using a 1-Butanol/Methanol extraction method before application to ESI-MS/MS analysis^2^, as described previously in detail (Alshehry et al., 2015; Bond et al., 2021). Quantification of lipids from MS analysis was performed using Mass Hunter Software (Agilent) and normalised to total phosphatidylcholine (PC). 249 lipid species were measured from 11 lipid classes, including: phosphatidylethanolamines (PE), Bis(9onoacylglycerol)phosphate (BMP), acylcarnitines (AC), hydroxy acylcarnitines (AC-OH), Cardiolipins (CL), lysophosphatidylcholines (LPC), lysoalkylphosphatidylcholines (LPC-O), lysoalkenylphosphatidylcholines (LPC-P), Lysophosphatidylethanolamines (LPE), lysoalkenylphosphatidylethanolamines (LPE-P), and Lysophosphatidylinositols (LPI). These lipid classes were measured due to previous studies which have implicated them in brain injury and their associations with mitochondrial metabolism, cellular membrane integrity, and cell death.

### Statistical analyses

Statistical analyses were performed with IBM’s SPSS version 27 software, with the exception of linear mixed models, analysed with R software. Before data analyses, Shapiro–Wilk and Levene’s tests were used to evaluate normality and homogeneity of variance, respectively, and non-parametric tests were used for data analyses if either assumption was violated (*p* > 0.05). To confirm injury after sham/ACHI procedures, video sign observation data was analysed with the non-parametric Friedman’s test. Beam traverse time (and traverse time Ln transformed) and hindlimb slip data was analysed with a one-way analysis of variance (ANOVA) repeated measures (injury and time as between- and within-subject effects, respectively) with the Greenhouse–Geisser correction employed. Beam slips were also analysed with Friedman’s test (corrected for multiple comparisons, alpha = 0.076). Linear mixed models were used to account for correlated measurement of hemispheric metabolites within the same animal, with Group, Hemisphere, and Group^*^Hemisphere entered into the model with subjects entered as a random term. The ROUT method of identifying outliers (*Q* = 1%) indicated that no outliers in the datasets. ETF and coupling assays were analysed using a two-way ANOVA (with injury and Complex/State as factors). RCR and lipidomic data was analysed using one-way ANOVAs. Multiple comparison Tukey *post hoc* tests were performed when main effects were statistically significant at *p* < 0.05. All data was presented as mean ± standard error of the mean (SEM).

## Results

### Repeated ACHI induced greater acute neurobehavioral deficits than singular and sham injuries

A video observation protocol was used to confirm injury in rats immediately after each procedure (*n* = 13–16/group; Figure 1B). Visible signs of injury were comparable between injuries for repeat ACHI rats, and for the final injury for repeat and single ACHI rats.

Parametric and non-parametric analyses were performed on beam traverse time and hindlimb slip data, which was evaluated as a measure of sensorimotor function at baseline, 1 h, and 4 h post-final injury (*n* = 13–16/group). One-way repeated measures ANOVA revealed a significant between-subject effect of injury [*F*(2, 41) = 15.84, *p* < 0.001] and within-subject effect of time [*F*(1.59, 64.99) = 10.34, *p* < 0.001] on the amount of hindlimb beam slips (Figure 1C), as well as an interaction effect of injury and time [*F*(3.17, 64.99) = 9.75, *p* < 0.001]. *Post hoc* analyses revealed that repeated ACHI rats displayed more slips than sham and single ACHI injury rats at 1 h post final injury (vs. sham: *p* = 0.006; vs. single ACHI: *p* = 0.009) and at 4 h post-final injury (vs. sham: *p* = 0.026; vs. single ACHI: *p* = 0.028). Beams slips were also analysed with Friedman tests, with alpha set to 0.017 to correct for multiple comparisons. This confirmed that there were no significant differences observed over time for sham rats (*p* = 0.758) and rats that received a single injury (*p* = 0.452); however, a significant difference was found over time within repeat injury rats (*p* = 0.003), with *post hoc* testing revealing increased slips when compared to baseline at 1 h (*p* = 0.005) and 4 h (*p* = 0.012).

For average time taken to traverse the beam (Figure 1D), one-way repeated measures ANOVA revealed a significant between-subject effect of injury [*F*(2, 41) = 14.60, *p* < 0.001] and within-subject effect of time [*F*(1.29, 52.74) = 6.39, *p* = 0.003], as well as an interaction effect of injury and time [*F*(2.57, 52.74) = 11.69, *p* < 0.001]. *Post hoc* analyses revealed that repeated ACHI rats took significantly longer to traverse the beam at 1 h and 4 h post final ACHI compared to sham (1 h: *p* = 0.019; 4 h: *p* < 0.001) and single ACHI (1 h: *p* = 0.012; 4 h: *p* = 0.002) rats. When the traverse time was Ln transformed to reduce positive skew, we found similar results, with a significant between-subject effect of injury [*F*(2, 41) = 15.04, *p* < 0.001] and interaction effect of injury and time [*F*(3.47, 71.13) = 10.82, *p* < 0.001], but no significant effect of time alone [*F*(1.74, 71.13) = 1.44, *p* = 0.243]. *Post hoc* testing confirmed that repeatedly injured rats took longer to cross the beam than sham and single injury rats at both 1 h and 4 h (*p* < 0.001).

### Repeated ACHI acutely altered some ^1^H-MRS metabolites

**Figure 2:**
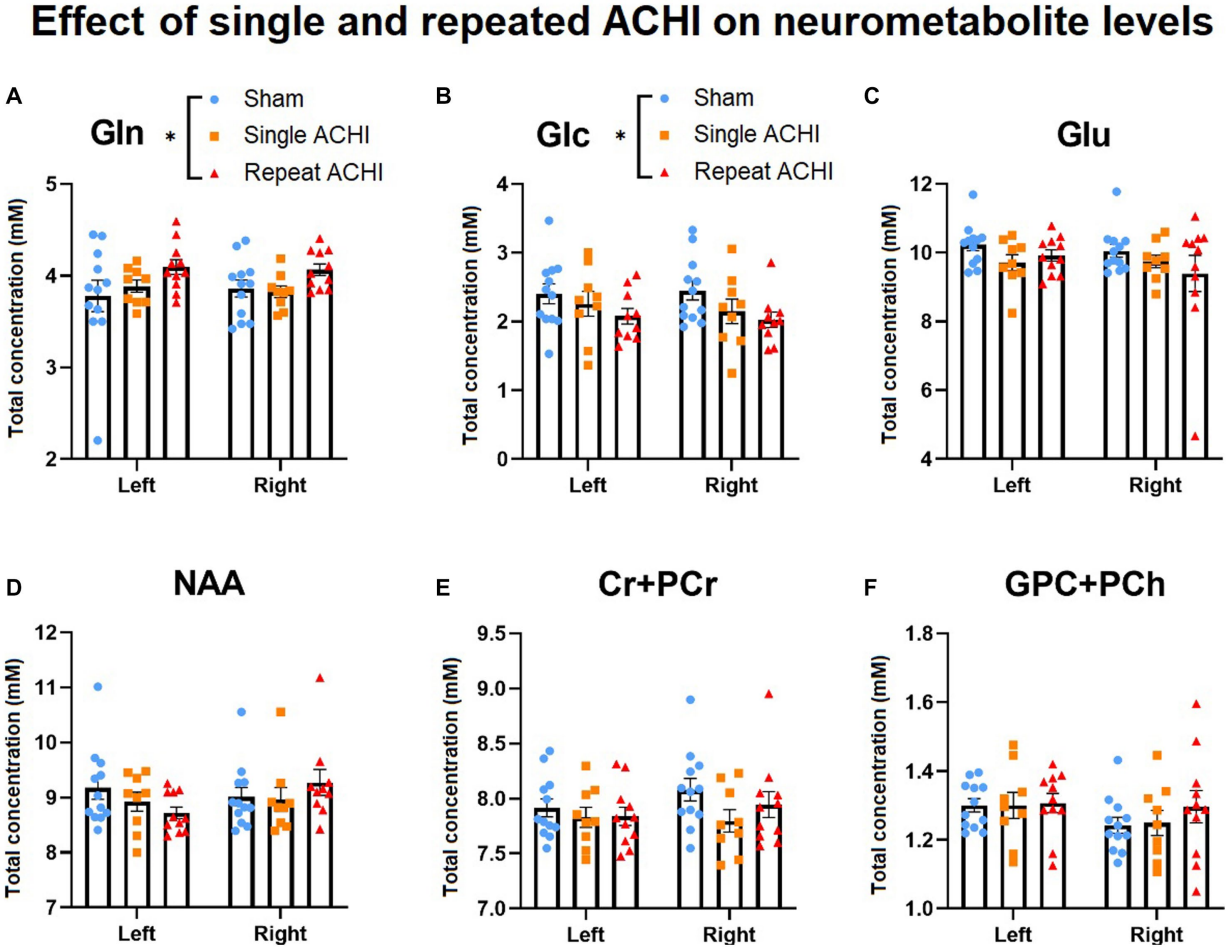
Effect of singular and repeated ACHI on neurometabolic concentrations. Repetitive ACHIs elevated Gln **(A)** and decreased Glc **(B)** 24 h post-final injury. No group differences were detected for Glu, NAA, Cr + PCr, GPC + PCh **(C)**–**(F)**. ^*^p < 0.05 sham vs. repeated injury. Data are presented as mean ± SEM.

^1^H-MRS was used to measure metabolite concentrations in both hemispheres of the hippocampus at 24 h post-final injury (*n* = 9–12/group; Figure 2). Main effects were analysed using a linear mixed model without an interaction term. We found a significant main effect of ACHI on levels of Gln in the repeat group [*t*(56) = 2.39, *p* = 0.020], but not the single group [*t*(56) = 0.31, *p* = 0.758], compared to sham. Tukey *post hoc* testing confirmed that repeat ACHI (*p* = 0.035), but not single ACHI (*p* = 0.981), increased Gln compared to sham rats (Figure 2A). Similarly, there was an effect of ACHI on Glc levels in the repeat group [*t*(56) = −2.23, *p* = 0.030], but not in the single group [*t*(56) = −1.15, *p* = 0.256]; *post hoc* analyses revealed a significant decrease in Glc after repeated ACHI (*p* = 0.029), but not single ACHI (*p* = 0.337), compared to sham (Figure 2B). ACHI had no effect on levels of Glu (*p* ≥ 0.113), NAA (*p* ≥ 0.467), Cr + PCr (*p* ≥ 0.388), or GPC + PCh (*p* ≥ 0.463; Figures 2C–F), but there was a significant effect of hemisphere for Cr + PCr [*t*(56) = 2.24, *p* = 0.029] and GPC + PCh [*t*(56) = −2.48, *p* = 0.016]. When an interaction term was introduced into the model, we found that there was no interaction effect of group^*^hemisphere on Gln (*p* ≥ 0.462) or Glc (*p* = 0.543), but there was an interaction effect of ACHI^*^hemisphere for NAA [*t*(56) = 3.13, *p* = 0.003] in rats that were injured repeatedly.

### ACHI had no effect on hippocampal mitochondrial bioenergetics

**Figure 3:**
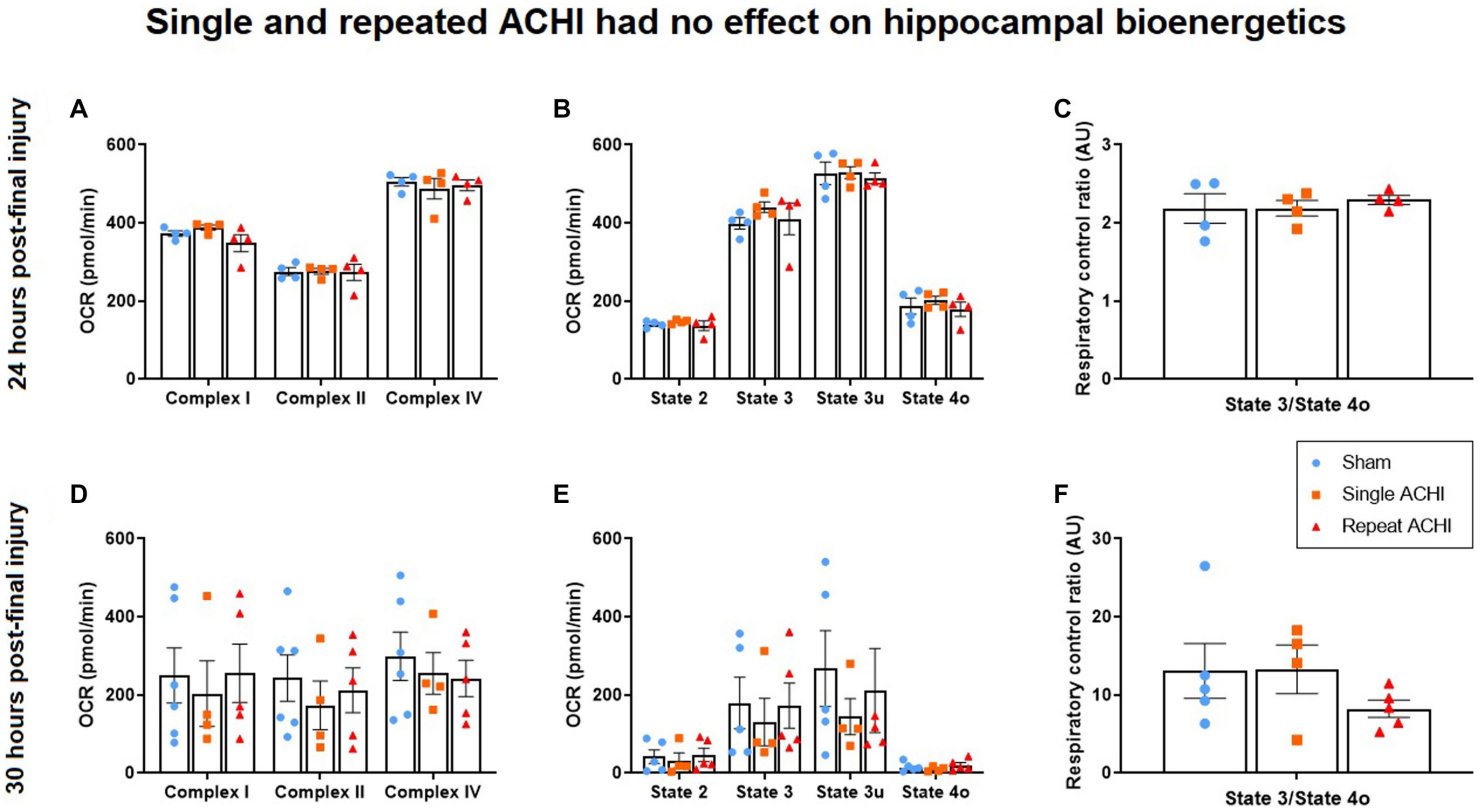
Effect of single and repeated ACHI on isolated hippocampal mitochondrial respiration. There were no group differences in the EFT assay, coupling assay, or RCR 24 h post-injury **(A)**–**(C)** or 30 h post-final injury **(D)**–**(F)**. Data are presented as mean ± SEM.

Hippocampal mitochondrial bioenergetics were assessed either 24 h post-final injury (without ^1^H-MRS) or after ^1^H-MRS at 30 h post-final injury (*n* = 4–6/group). We did not observe any significant effect of ACHI in the ETF assay (Figures 3A–D; *p* ≥ 0.307), coupling assay (Figures 3B–E; *p* ≥ 0.435) or RCR at 24 h post-injury (Figure 3C; *p* = 0.792). However, there was a significant effect of ACHI on RCR at 30 h post-injury (Figure 3F; *p* = 0.037), although no group differences were found with post-hoc testing (sham vs. repeat: *p* = 0.072). The mitochondrial bioenergetic measures were more variable for rats that were assessed at 30 h post-injury, which may be related to unavoidable variables like combining multiple cohorts of rats for analysis and varied resting times after scans prior to tissue collection.

### Repeated ACHI altered some lipidomic profiles in the cortex but not the hippocampus

**Figure 4:**
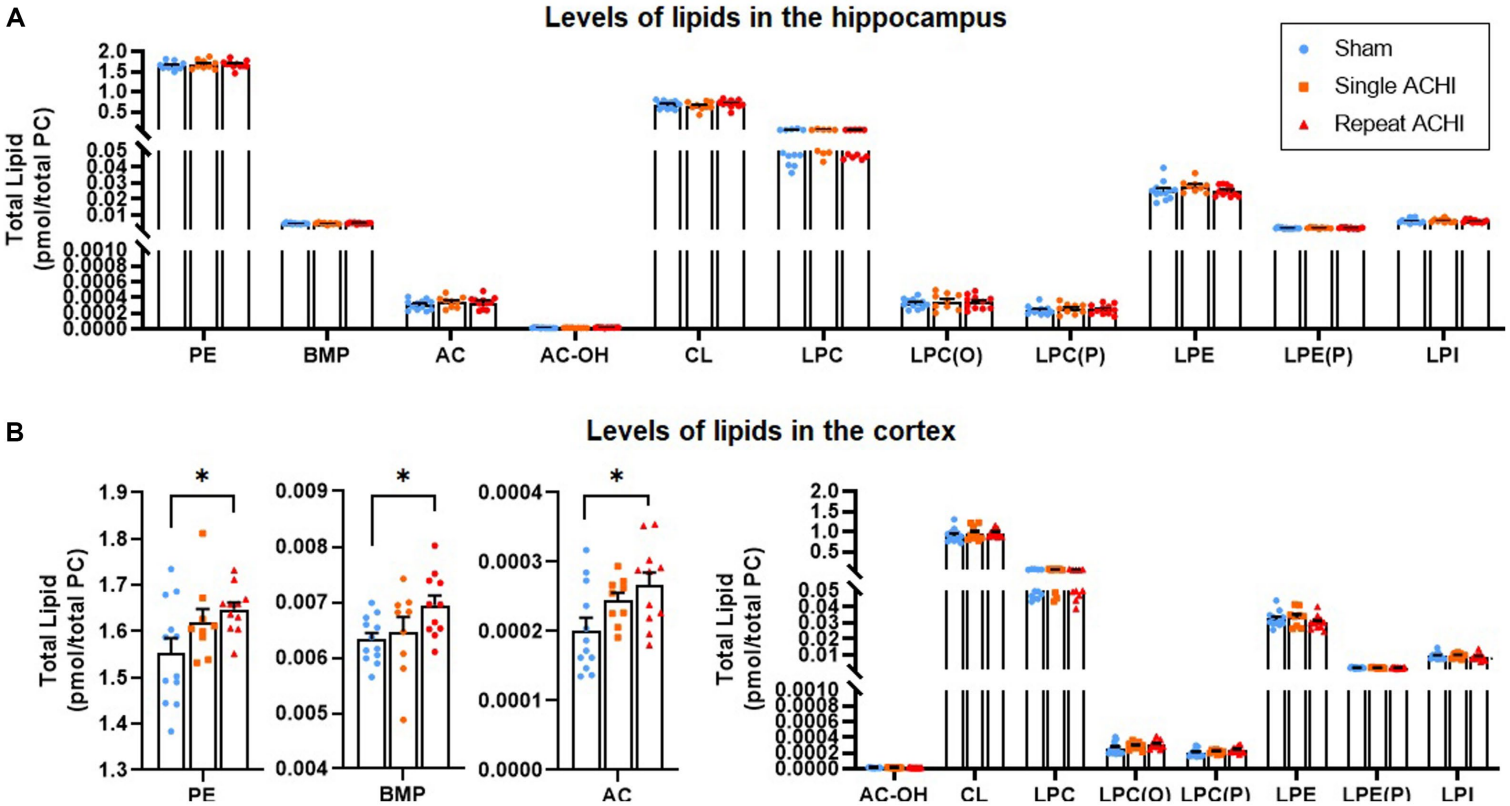
Effect of single and repeated ACHI on lipidomics in the hippocampus and cortex. There were no group differences in the hippocampus **(A)** but PE, AC, and BMP were elevated after repeated ACHIs in the cortex **(B)**. ^*^p < 0.05 sham vs. repeated injury. Data are presented as mean ± SEM.

239 lipid species were measured over 11 lipid classes, which have previously been associated with brain injury or have been shown to play a role in mitochondrial metabolism, cellular membrane integrity, and cell death. Lipidomic evaluations were carried out first in the hippocampus, but no significant group differences were observed for any lipid of interest (one-way ANOVAs: *p* ≥ 0.238; Figure 4A). We proceeded with a lipidomic evaluation of the cortex (Figure 4B), because region-specific alterations in lipids that have been linked to brain injury have been observed in the past (Hogan et al., 2018; Hubbard et al., 2019). In the ipsilateral cortex, we found a significant effect of ACHI on levels of PE [*F*(2, 31) = 3.66, *p* = 0.038], AC [*F*(2,31) = 4.27, *p* = 0.024], and BMP [*F*(2, 31) = 3.41, *p* = 0.047], but not AC-OH, CL, LPC, LPC(O), LPC(P), LPE, LPE(P), or LPI (*p* ≥ 0.113). Rats exposed to repeated ACHIs had higher levels of PE, AC, and BMP compared to rats that had a single ACHI and significantly more than sham rats (PE: *p* = 0.036; AC: *p* = 0.020; BMP: *p* = 0.044).

## Discussion

The main findings of this report provide evidence that repeated mTBIs produce worse neurobehavioral outcomes than a single mTBI, and that these outcomes are accompanied by alterations in multiple neurometabolites and lipids. First, we confirmed concussive-like injuries for all ACHIs with a clinically relevant video sign observation protocol; however, only rats given repeated mTBIs had deficits in the sensorimotor beam-walk task at 1- and 4 h post-final injury. Secondly, with ^1^H-MRS at 24 h, we found that concentrations of Gln and Glc were altered by repeated mTBI. Although Gln and Glc are involved in mitochondrial bioenergetics, we did not find evidence of impaired hippocampal mitochondrial respiration in these conditions. Finally, although single and repeated mTBI did alter lipid levels in the hippocampus, more notable repeated mTBI effects were found in the cortex.

In the present study, we used the well-characterized ACHI mTBI model, which consistently produces neurobehavioral impairments in the absence of skull fracture, significant focal contusion, or other complications that can occur in other animal models of mTBI (Shultz et al., 2017; Christie et al., 2019). Video observations confirmed that ACHI was associated with signs of brain trauma (e.g., lying motionless, seizures, absence of reflexes, etc.). We also found that sensorimotor deficits, as measured by the time it took to cross a beam and the number of hindlimb slips while crossing the beam, persisted 1- and 4 h after the final injury in rats that received repetitive injuries but not a single injury. These findings provide construct validity for the ACHI model considering that clinical studies show that mTBIs can cause prolonged subtle gait and balance impairments (Howell et al., 2017; Lynall et al., 2019).

Mounting evidence suggests that alterations in neurometabolite concentrations, due to factors induced by trauma, such as disruption of neuronal membranes, axonal stretching, and ion channel dysregulation, are implicated in the neurobehavioral sequalae of mTBI (Giza and Hovda, 2014; Kierans et al., 2014). As such, the evaluation of a panel of neurometabolites with ^1^H-MRS has been postulated to have clinical utility as a diagnostic and prognostic biomarker for individuals with suspected concussion (Gasparovic et al., 2009; Vagnozzi et al., 2010; Chamard et al., 2013; Gardner et al., 2014; Veeramuthu et al., 2018; Thomas et al., 2022). Corroborating this notion, pre-clinical studies show that single (Xu et al., 2011; Lyons et al., 2018) and repeated (Wright et al., 2016) brain injuries can acutely alter some neurometabolite concentrations observed with ^1^H-MRS. Here we evaluated the effect of single and repeated ACHIs on the concentration of several neurometabolites that have been implicated in mTBI, namely Glu, Gln, Glc, NAA, Cr + PCr, and GPC + PCh (Shutter et al., 2004; Yeo et al., 2011; Veeramuthu et al., 2018).

Due to the similarity of the molecular structures of Glu and Gln, resulting in similar peaks on the spectra, early clinical studies traditionally combined the absolute concentrations of Glu and Gln (Ashwal et al., 2004; Shutter et al., 2004; Yeo et al., 2011; Wright et al., 2016). This method does not allow researchers to distinguish between Glu and Gln, which may undergo opposite directions of concentration change, but recent technological improvements now make it possible to evaluate Glu and Gln separately (Ramadan et al., 2013). We did not find significant group differences in the concentration of Glu with ^1^H-MRS, but we did observe a marked increase of Gln in the hippocampus 24 h after repeated but not singular mTBI. This may be due to the astrocytic uptake of excess Glu that is released acutely in response to TBI (Pellerin and Magistretti, 1994; Dorsett et al., 2017) and subsequently converted and stored as Gln to help maintain metabolite homeostasis (Ramadan et al., 2013; Guerriero et al., 2015). Future studies are required to test this hypothesis and to further understand the implications that single and repeated mTBI have on altered glutamatergic metabolism, which is linked to various pathological conditions (Allen et al., 2021). This study, to the best of our knowledge, was the first to detect a reduction in hippocampal Glc using ^1^H-MRS following repeated mTBI in rodents. This decrease may be explained by a reduction in Glc uptake, as observed in several animal and human TBI studies (Yoshino et al., 1991; O’Connell et al., 2005; Peskind et al., 2011), or perhaps due to hyper-glycolysis (i.e., increased breakdown of cellular stores of Glc to produce ATP) to compensate for potential ATP depletion that can occur after TBI (Yoshino et al., 1991). However, additional research is required to confirm the presence of hyper-glycolysis after single and repeated mTBI and elucidate whether this contributes to the reductions in Glc levels observed in mTBI models.

We did not detect alterations in the hippocampal concentrations of NAA, Cr + PCr, or GPC + PCh between injury groups. These findings are consistent with some clinical (Maugans et al., 2012; Schranz et al., 2018) and rodent (Xu et al., 2011; Wright et al., 2016) studies; however, NAA may represent a potential biomarker of injury severity (Vagnozzi et al., 2008; Veeramuthu et al., 2018). Our results could differ from clinical studies that report a reduction in NAA/Cr following single mTBI (Veeramuthu et al., 2018), repeated mTBI (Vagnozzi et al., 2008), and in athletes with Persistent Post-Concussive Symptoms (Bartnik-Olson et al., 2014) for several reasons, including differences in species, sex, voxel size and positioning, protocol and analysis method. The current study focused on hippocampal gray matter, but other preclinical and clinical studies that evaluated white matter regions (e.g., corpus callosum and frontal lobes) have found lower levels of Cr + PCr in mTBI patients compared to controls with no differences between groups in gray matter (Gasparovic et al., 2009), indicating that metabolic response to mTBI may vary between brain regions. We also found no differences in GPC + PCh, which was consistent with a previous study of repeated mTBI in rats (Wright et al., 2016). There are many other possible metabolites that could be involved in mTBI neurobehavioral sequalae that should be evaluated in this and other brain injury models, and in males and females, who respond differently to mTBI (Wright et al., 2017, 2022). The underlying neuropathology causing potential metabolite alterations identified with ^1^H-MRS is poorly understood; further research efforts are required if we are to better understand these mechanisms, which could lead to the discovery of therapeutic targets and biomarkers of brain injury.

Although mTBI is thought to lead to a period of increased cerebral vulnerability that may be contributed by a dysregulation of bioenergetics (Lyons et al., 2018), the association between deficient mitochondrial respiration and metabolic alterations induced by mTBI is poorly understood. Considering that Gln and Glc, along with other metabolites we evaluated, are highly involved in mitochondrial respiration (Yetkin-Arik et al., 2019; Shaghaghi et al., 2021), and that targeting the mitochondria provides protection against secondary damage (Stelmashook et al., 2019), we investigated whether impairments in mitochondrial bioenergetics were present in these conditions. We focused on the ipsilateral hippocampus as this region has been found to be affected in the ACHI model, with evidence of increased glial reactivity and alterations in several proteins associated with energy metabolism (Pham et al., 2019). Moreover, other mTBI studies have reported changes in hippocampal proteins related to energy metabolism (Opii et al., 2007; Bolton and Saatman, 2014; Girgis et al., 2016). Mitochondrial dysfunction is also observed in disorders that are highly comorbid with mTBI, such as emotional and cognitive disorders (Allen et al., 2018; Lejri et al., 2019). However, we found no changes in OCRs of ETC complexes I, II, and IV, or mitochondrial respiration States 2, 3, 3u, and 4o, 24 h after single and repeated mTBI. Furthermore, there were no significant findings in the RCR, indicating that mitochondrial function was not altered between groups at 24 h post-final ACHI (Brand and Nicholls, 2011). It is possible that mitochondrial bioenergetics would be altered by single and repeated mTBI at later timepoints, considering that other groups have found reduced hippocampal mitochondrial respiration 48 h post-final injury after either 4, 7, or 10 weight-drop concussions (Severo et al., 2020), and 28 days after a single closed-head injury (Lyons et al., 2018). In line with this, another group reported that a single closed-head injury reduced hippocampal State III mitochondrial respiration 48 h post-injury, but not 6- or 24 h post-injury (Hubbard et al., 2019). They also found that mitochondrial respiration was decreased at 24- and 48 h post-injury in the cortex – suggesting that concussions may induce region-specific deleterious effects – and a second injury prolonged cortical mitochondrial impairment (Hubbard et al., 2019). Our results may also differ from other studies because of differences in injury method, impact velocity, species, anaesthetic protocols, surgery, and head restraint (Satchell et al., 2005; Picard et al., 2018; Shen et al., 2019; Salman et al., 2021). It is also important to consider the young age of the animals in this study, given that age influences response to and recovery from brain trauma (Tremblay et al., 2019), which may be tied to bioenergetic and lipidomic factors (Gilmer et al., 2010). It could be that mitochondrial functions become compromised after a threshold of cellular “wear and tear” (i.e., allostasis) has been reached – which may naturally occur with age (Allen et al., 2021) – compromising energy production, metabolism homeostasis, and mitochondrial membrane integrity, leading to mTBI neuropathology (Satchell et al., 2005; Picard et al., 2018; Shen et al., 2019; Salman et al., 2021). Notably, we observed more variable functionality in the ETC complexes and mitochondrial states of respiration in rats that were analysed 30 h post-final injury compared to rats that were analysed at 24 h post-injury. This is likely due to the limited number of rats able to be scanned per day necessitating multiple mitochondrial assays, with inter-assay variability apparent. It is also possible that varied resting times after scans before tissue collection, and subsequently, increased time between first and last tissue collection, were a factor. It would be valuable to assess mitochondrial function in the cortex after ACHIs, considering that lipid imbalances were more pronounced in the cortex than the hippocampus.

Region-specific lipid alterations induced by mTBI likely depend on the nature of the injury and its pathophysiological consequences. We found that mTBI-induced lipid alterations were more pronounced in the cortex – where repeated ACHIs elevated PE, AC, and BMP – than in the hippocampus, where no significant group differences were observed. These findings are consistent with a closed-head mTBI study that reported elevations in cortical PE levels 24 h after repeated mTBI in Tau transgenic mice, but not in the hippocampus. Notably, this study also found that PE levels had normalized 3- and 12 months post-injury (Ojo et al., 2018). However, in contrast, they also found that repeated mTBI decreased LPE and LPC levels at 24 h post-injury in the hippocampus, but not in the cortex, where LPE and LPC were elevated 3 months post-final injury (Ojo et al., 2018). Work with various TBI models suggests that PE is elevated in the acute phase (i.e., 24 h post-injury) but not in the chronic phase after an injury (Abdullah et al., 2014; Emmerich et al., 2017; Hahnefeld et al., 2022). This could also be true for LPE, LPC, and other lipids (Hahnefeld et al., 2022). Our results are in line with clinical reports that have detected higher levels of PE in cerebrospinal fluid as early as 1 day post-TBI (Pasvogel et al., 2008); however, PE levels were lower by day 6 post-injury (Pasvogel et al., 2010), further demonstrating the time-sensitive nature of lipid alterations. It is thought that heightened levels of PE may be indicative of compromised membrane integrity (Yoon et al., 2022). We also found that repeated mTBIs heightened AC levels in the cortex, which is consistent with another study that reported heightened AC levels in the developing brain after a lateral fluid-percussion injury (Chitturi et al., 2019), and a clinical study that reported higher serum AC levels is association with poorer functional outcomes 1 and 6 months post-TBI in patients with intracranial injury (Lee et al., 2023). It is possible that accumulations of AC are mediated by mitochondrial dysfunction (McGill et al., 2014) and contribute to the inflammatory response following brain trauma (Rutkowsky et al., 2014). BMP, which regulates endosomal-lysosomal trafficking and serves as a docking structure for enzymes involved in sphingolipid breakdown, is important for the integrity and function of lysosomes and has been implicated in the inflammatory response and neurodegenerative disorders (Showalter et al., 2020), which are more likely to develop after TBIs (Brett et al., 2022). BMP was increased by repeat mTBI in these conditions, but it is unclear whether alterations in BMP underlie neurobehavioral alterations or represent a biomarker of mTBI pathology. Although speculative, the accumulation of PE, BMP, and AC in the cortex but not the hippocampus may suggest that cortical mitochondria are more vulnerable after mTBI. However, cortical lipids may be altered more than hippocampal lipids due to the increased likelihood of cortical vs. hippocampal damage from the direct impact and consequent movement of the brain inside the skull. More research is also required to elucidate how imbalances in lipid metabolism, signalling, and transport influence the progression of mTBI pathology.

There are several limitations in this study. First, only male rats were included, even though evidence suggests that there are sex-based differences in various aspects of the response to mTBI (Wright et al., 2017, 2021, 2022), including neurometabolite concentrations (Lyons et al., 2018) and mitochondrial dysfunction (Kalimon and Sullivan, 2021). As such, further investigation into possible sex-based changes in metabolic and lipidomic pathology in response to single and repeated mTBI is warranted, especially considering that sex hormones have neuroprotective properties (Wei and Xiao, 2013). Although the ACHI procedure features an unrestrained head, the foam platform and piston extension may limit the degree of head rotation when compared to other models, such as the lateral impact and Closed-Head Impact Model of Engineered Rotational Acceleration models. Another limitation is that we did not account for mitochondria number, which could influence overall ATP production. There is a possibility that changes in mitochondrial number may consequently affect Gln/Glc levels due to altered whole cell capacity. It should also be emphasized that whilst a single mTBI did not induce sensorimotor deficits or alter levels of metabolites, lipids, or mitochondrial bioenergetics, evidence of a concussive like injury was provided by acute neurological observations, and a consistent elevation in serum NfL in a subset of these rats presented previously (O’Brien et al., 2021). However, assessments of hippocampal function (e.g., learning and memory) as an additional measure of injury severity at each timepoint would have been beneficial, and whether alterations in neurometabolites correlated with cognitive impairment. Furthermore, our neurometabolic data is limited to one acute timepoint, although patterns of expression may change over time. Future studies could determine the persistency of concussive symptoms, including learning and memory deficits, as well as related neurometabolic and lipidomic alterations.

## Conclusion

Alterations in metabolites Gln and Glc were observed after repeated mTBI in the hippocampus using ^1^H-MRS, but these alterations were not associated with hippocampal mitochondrial dysfunction. Lipids PE, BMP, and AC were elevated in the cortex after repeated mTBI, but not the hippocampus. Our data supports the fact that repeated mTBIs in short succession can have cumulative effects, evidenced by the poorer performance in sensorimotor tests when rats received repeated vs. singular injuries, as well as greater neurometabolic and lipidomic abnormalities. More research is needed to understand the underpinning neuropathological changes responsible for these alterations.

## Data availability statement

The raw data supporting the conclusions of this article will be made available by the authors, without undue reservation.

## Ethics statement

The animal study was reviewed and approved by Alfred Research Alliance Animal Ethics Committee.

## Author contributions

LP, SB, SS, BD, and SM contributed to the study design. LP, SB, WO’B, SS, BD, DW, and SM contributed to data collection. JA completed data analysis and drafted the manuscript. GS assisted data analysis. All authors contributed to the article and approved the submitted version.

## Funding

This work was supported by the National Health and Medical Research Council to DW [grant number: 1174040] and SS [grant number: 1141643].

## Conflict of interest

The authors declare that the research was conducted in the absence of any commercial or financial relationships that could be construed as a potential conflict of interest.

## Publisher’s note

All claims expressed in this article are solely those of the authors and do not necessarily represent those of their affiliated organizations, or those of the publisher, the editors and the reviewers. Any product that may be evaluated in this article, or claim that may be made by its manufacturer, is not guaranteed or endorsed by the publisher.
